# The Observed Association between Maternal Anxiety and Adolescent Asthma: Children of Twin Design Suggest Familial Effects

**DOI:** 10.1371/journal.pone.0066040

**Published:** 2013-06-12

**Authors:** Ida Havland, Cecilia Lundholm, Paul Lichtenstein, Jenae M. Neiderhiser, Jody M. Ganiban, Erica L. Spotts, Hasse Walum, David Reiss, Catarina Almqvist

**Affiliations:** 1 Department of Medical Epidemiology and Biostatistics, Karolinska Institutet, Stockholm, Sweden; 2 Department of Psychology, The Pennsylvania State University, University Park, Pennsylvania, United States of America; 3 Department of Psychology, George Washington University, Washington, District of Columbia, United States of America; 4 Division of Behavioral and Social Research, National Institute on Aging/National Institutes of Health, Bethesda, Maryland, United States of America; 5 Yale Child Study Center, Yale School of Medicine, New Haven, Connecticut, United States of America; 6 Department of Women’s and Children’s Health and Astrid Lindgren Children’s Hospital, Stockholm, Sweden; Ludwig-Maximilians-University Munich, Germany

## Abstract

**Background:**

Previous studies indicate that maternal anxiety is associated with asthma in the adolescent child, but mechanisms are unclear.

**Objective:**

To investigate the association between maternal anxiety and maternal, self- and register-based report of asthma in the adolescent child, and whether the association remains after control of familial confounding (shared environmental and genetic factors).

**Method:**

From the Twin and Offspring Study of Sweden, 1691 mothers (1058 twins) and their adolescent child were included. The association between maternal self-reported anxiety (Beck Anxiety Inventory (BAI) and Karolinska Scales of Personality (KSP) somatic or psychic anxiety) and asthma based on subjective (maternal or child report) or objective (register-based diagnosis and medication) measures were analysed using logistic regression. The children-of-twins design was used to explore whether genes or environment contribute to the association.

**Results:**

Maternal BAI anxiety (OR 2.02, CI 1.15–3.55) was significantly associated with adolescent asthma reported by the mother. Maternal KSP somatic anxiety (OR 1.74, CI 1.04–2.91) and psychic anxiety (OR 1.74, CI 1.05–2.86) was significantly associated with breathlessness reported by the adolescent child. In contrast, maternal anxiety was not associated with increased risk for the register-based outcomes of asthma diagnosis or medication. The results remained also after adjusting for covariates and the children-of-twins analyses which indicate that the association was due to familial confounding.

**Conclusions:**

We found some associations between maternal anxiety and subjectively reported offspring asthma or breathlessness which may be due to familial effects. A likely candidate for explaining this familial confounding is heritable personality traits associated with both anxiety and subjective measures of asthma.

## Introduction

Asthma is one of the most common chronic diseases in childhood [1]. Several cross-sectional and longitudinal studies indicate that maternal anxiety is associated with asthma; however the underlying mechanisms that account for this association are poorly understood [2], [3], [4], [5], [6]. For example, there are some reports that childhood asthma causes anxiety [7] in the caregiver. Conversely, epidemiological studies have suggested that maternal anxiety during pregnancy [4], [8], [9], [10] and during the postpartum period are predictive of development of asthma in young children [2], [9], [11], [12], [13], [14]. Proposed mechanisms include changes in gene expression in susceptible genes that regulate the immune-response and Hypothalamic-Pituitary-Adrenal axis [15], [16], [17].

On the other hand, maternal anxiety and children’s asthma may not be causally related. For example, associations between asthma and anxiety may be due to environmental stressors in the family (shared environment), factors that are held in common by the adult parents (non-shared environment) or parent-child associations due to genes that affect anxiety in the parent and asthma in the child. The children-of-twin design offers a possibility of studying whether associations between parental characteristics and child outcomes are causal or due to confounding from genes or familial environment [18]. Associations may also reflect rater bias. In most previous studies, mothers reported on their own anxiety and their adolescent children’s asthma symptoms [2], [3], [6], [7], [14]. The use of a single rater for both constructs could lead to overestimation of the association between maternal anxiety and child asthma. The effect of maternal anxiety on child asthma may be studied also for self-reported and objectively measured asthma diagnosis or medication in national health registers.

In summary, although some findings support cross-sectional associations between maternal anxiety and asthma in the adolescent child [2], [3], [7], [19], mechanisms of these associations is unclear.

The primary aim of this study is to investigate the mechanism that underlies links between maternal anxiety and asthma in the adolescent offspring. Specifically, the children-of-twins design was used to address the issue of whether an association between maternal anxiety and offspring asthma is caused by family-wide, environmental and/or genetic factors. The current study is also unique because it utilises child reports and objective measures of asthma, enabling us to address the issue of rater bias.

## Methods

### Study Population

**Figure 1:**
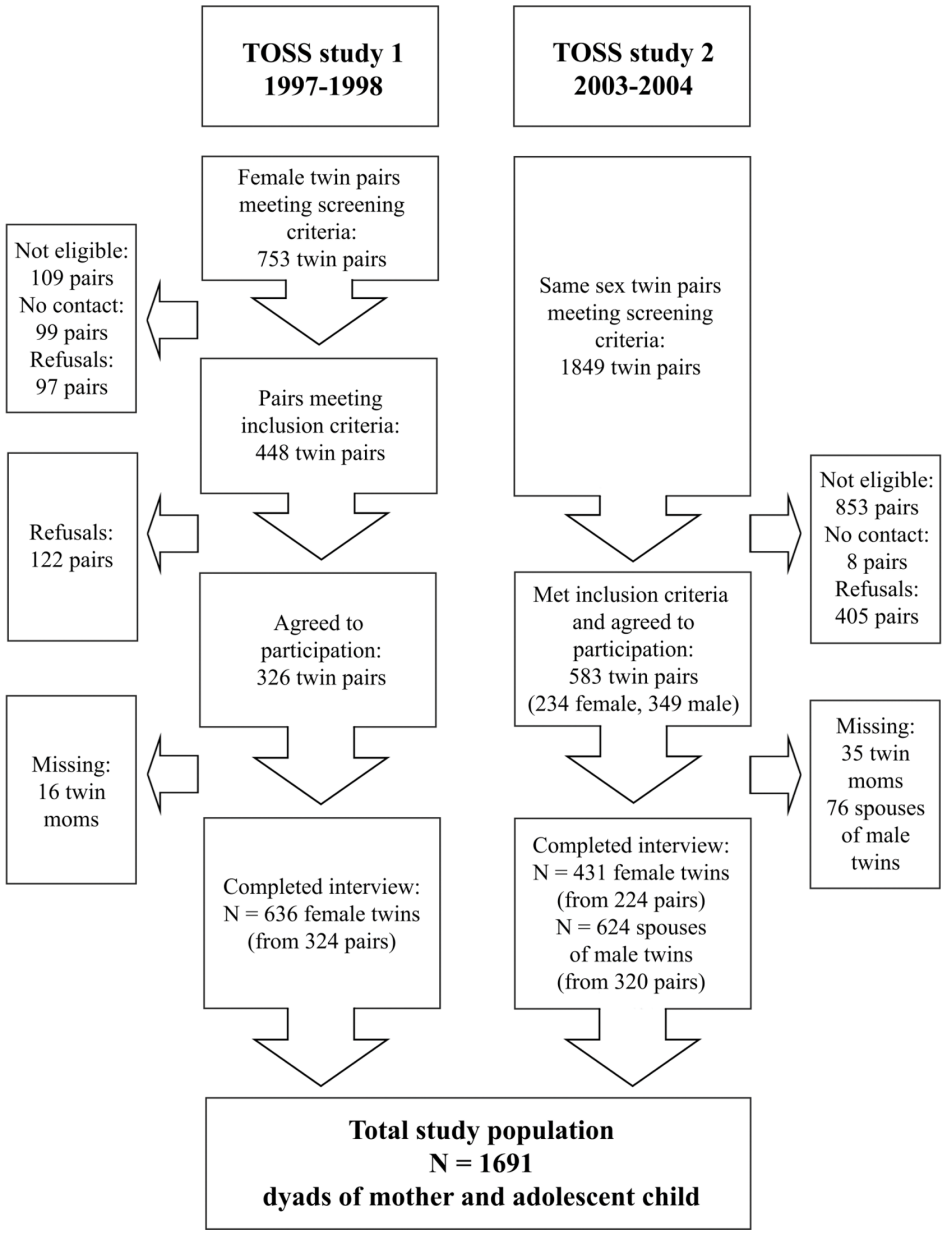
Flow chart of the cross-sectional TOSS studies one (1997–1998) and two (2003–2004).

The Twin and Offspring Study of Sweden (TOSS) is a two parts cross-sectional study based on questionnaires, that was conducted 1997–1998 (study 1) and 2003–2004 (study 2) with a focus on genetic and environmental influences on parenting and inter-familiar relationships [20].The TOSS study population was derived from the Swedish Twin Registry, which contains more than 195 000 twins born in Sweden since 1885 [21]. The first step was to identify twins from same sex pairs, where both twins had an adolescent child registered at the same address as the parents (screening criteria). The children of each twin in a pair had to be of the same sex and could not be more than four years apart in age. In a second step those twins were contacted to see if they fulfilled the inclusion criteria. Those were 1) having a long-term spouse (defined by living more than five years together) and 2) confirmation that the targeted child was living at home. In total, 909 same-sex twin-pairs were included together with their spouses, and the targeted adolescent child of each twin (Figure 1). In study 1 only same sex female twin pairs were included, while in study 2 also male same sex pairs were included. Of the twin-pairs 254 were MZ female, 285 DZ female, 128 MZ male and 183 DZ male. To maximize our sample, analyses for the current study both included female twins and the female spouses of male twins. Data were collected by questionnaires completed by mothers and their adolescents as well as from population based registers regarding inpatient and outpatient specialist care and dispensed prescribed drugs.

### Maternal Anxiety

Maternal anxiety was reported in questionnaires by two different measures at the time of survey administration when the children were 11–22 years of age. From the Karolinska Scales of Personality (KSP) the sub-scales on somatic anxiety and psychic anxiety were used. They attempt to measure a person’s qualities in temper and mood in general. These scales have been validated [22]. Beck Anxiety Inventory (BAI) was also used to assess anxiety during the last week [23]. The KSP was included in the questionnaires for both studies, while BAI only was included in study 2. If an item was missing the mean of the scores of the other items in the measure was imputed. If more than one item was missing the dyad was excluded from the analyses of that measure. The scales were analysed continuously or categorized into approximate tertiles for analyses.

### Outcome Variables

Children’s asthma symptoms and diagnosis were derived from single items taken from the Child Behavior Check list (CBCL) [24] and the Physical Symptoms Inventory (PSI) [25]. The CBCL primarily measures the child’s health but also includes specific questions on somatic illness pertinent to asthma symptoms. Maternal report of asthma in the child was denoted if the question “Has asthma” was answered with “somewhat or sometimes true” or “very true or often true” and self-reported asthma in child was denoted if “I have asthma” was answered with the above. In addition, a single item related to wheezing (breathlessness) was taken from the Physical Symptoms Inventory (PSI). The CBCL-scale was included in both TOSS studies, but PSI only in study 2. Asthma outcome was also measured objectively as prescribed asthma medication from the Prescribed Drug Register (PDR) (at least two purchases of asthma drugs with ATC-codes R03AC, R03AK, R03BA or R03DC) since the start of the register July 1 2005 until Dec 31 2009, and as a diagnosis of asthma ever registered in the National Patient Register (PAR) until Dec 31 2009 (ICD-9∶493 and ICD-10: J45–J46). In PDR, all prescribed drugs dispensed at pharmacies in Sweden are registered since the start. PAR is a Swedish national register for inpatient (start in 1964, full geographic coverage since 1987) and outpatient (since 2001) specialist care. Sweden offers free inpatient and outpatient medical care to all residents. The National Patient Register covers inpatient and outpatient visits, but not visits to general practitioners and therefore we included asthma medication use from the Prescribed Drug Register as an additional asthma ascertainment strategy in the present analyses. By using these asthma ascertainment criteria, we believe that we were able to identify the vast majority of asthma cases that had come to the notice of the health care system. The kappa measure of agreement between maternal-reported asthma in child, self-reported asthma, asthma diagnosis and asthma medication in child were good or moderate, whereas the agreement for self-reported breathlessness and the others measures was poor.

### Covariates

Covariates that were available from the maternal questionnaires were the child’s sex, birth year as well as mother’s smoking and the twins’ zygosity. From national registers we collected data on birth weight, preterm birth (<36 weeks), caesarean section and maternal smoking during pregnancy (Medical Birth Register), maternal education at time of study (Educational Register), maternal asthma diagnosis from PAR and asthma medication (at least two records) from PDR, in order to adjust for potential confounders.

### Statistics

Odds ratios (OR) and 95% confidence intervals (CI) were used to assess the association between maternal anxiety and the asthma outcomes. A Generalized Estimating Equation model with the binomial distribution and the logit link was utilised, with adjustment for correlation within twin pairs. In order to explore the possibility of familial confounding a children-of-twins approach [18] was conducted in children where the mother herself was a twin and where the twin sister also participated in the study. Thus the children’s outcomes were analyzed both with regard to their own mothers’ anxiety and with regard to their aunts’ anxiety. If the children’s asthma was associated with their aunts’ anxiety, it can be inferred that family effects (genetic factors or environmental factors shared by the female co-twins) contributes to the associations between maternal anxiety and child asthma. The complete case approach was used in all analyses and performed in SAS v. 9.3. Results were considered significant if the p-value was below 0.05.

### Ethical Considerations

Permission for the study was obtained from the Regional Ethical Review board in Lund and Stockholm, Sweden. The data is archived at the Department of Medical Epidemiology and Biostatistics at Karolinska Institutet. Meta-data has been submitted to the Swedish National Data Service SND.

## Results

Table 1 displays descriptive data of the study population, which includes all female twins in studies 1 and 2 as well as female spouses of male twins from study 2. Study 1 only invited female twins, while 59% of mothers in study 2 were spouses of twins and therefore did not have any reported zygosity. Of the female twins in both cohorts, 47.2% were MZ. There was no major difference in distribution of children’s sex between the two cohorts (49.1% male in study 1 and 52.6% in study 2), and the cohorts were similar for most background variables except birth year of the child (born earlier in study 1), maternal smoking (more prevalent in study 2), and maternal education (lower in study 2).

**Table 1:** Descriptive data of the study population.

		Study 1A, C 1997–1998				Study 2B, C 2003–2004	
		n		%		n	%
**Background:**							
Study population			636			1055	
Sex of Child	Male		312		49.1	555	52.6
	Female		324		50.9	500	47.4
Birth year, child	1976–1979		115		18.1	0	0.0
	1980–1984		413		64.9	100	9.5
	1985–1989		108		17.0	615	58.3
	1990–1993		0		0.0	340	32.2
	Missing		0		0.0	0	0.0
Age, child	Mean (range)		15.5 (11–21)			15.9 (11–22)	
Birth weight	<2500 g		16		2.5	36	3.4
	≥2500 g		614		96.5	1011	95.8
	Missing		6		1.0	8	0.8
Preterm birth (<36 weeks)	No		616		96.9	1022	96.9
	Yes		14		2.2	27	2.6
	Missing		6		1.0	6	0.6
Caesarean section	No		550		86.5	951	90.1
	Yes		82		12.9	99	9.4
	Missing		4		0.6	5	0.5
Maternal smoking during	No		234		36.8	780	73.9
pregnancy^1^	Yes		71		11.2	198	18.8
	Missing		331		52.0	77	7.3
Birth year, mother	1943–1949		128		20.1	23	2.2
	1950–1954		244		38.4	117	11.1
	1955–1959		211		33.2	327	31.0
	1960–1964		46		7.2	400	37.9
	1965–1973		2		0.3	181	17.2
	Missing		5		0.8	7	0.7
Maternal smoking at study	No		512		80.5	869	82.4
date	Yes		121		19.0	177	16.8
	Missing		3		0.5	9	1.0
Maternal asthma	No		570		89.6	963	91.3
	Yes		60		9.4	88	8.3
	Missing		6		1.0	4	0.4
Maternal education	Compulsory school, 9 yrs		76		11.9	75	7.1
	Upper secondary school		307		48.3	548	51.9
	College/university		252		39.6	426	40.4
	Missing		1		0.2	6	0.6
Maternal twin relation	Monozygotic twin		292		45.9	207	19.6
	Dizygotic twin		344		54.1	215	20.4
	Partner is twin		0		0.0	612	58.0
	Missing		0		0.0	21	2.0
**Maternal anxiety:**							
Karolinska Scales of Personality (KSP):							
Somatic anxiety	10–12	157		24.7		355	33.6
	13–17	242		38.0		395	37.4
	18–36	231		36.3		298	28.4
	Missing	6		1.0		7	0.7
	Median (Q_1_; Q_3_)	16 (13;19)				14 (12;18)	
Psychic anxiety	10–17	230		36.2		386	36.6
	18–22	204		32.1		340	32.2
	23–39	198		31.1		322	30.5
	Missing	4		0.6		7	0.7
	Median (Q_1_; Q_3_)	20 (16; 24)				20 (16; 24)	
Beck Anxiety Inventory (BAI):							
	0–1	NA				322	30.5
	2–5	NA				378	35.8
	6–47	NA				350	33.2
	Missing					5	0.5
	Median (Q_1_; Q_3_)	NA				3 (1; 7)	
**Child’s asthma/asthma symptoms:**							
Maternal report of Asthma	No	562		88.3		958	90.8
in Child	Yes	56		8.8		93	8.8
	Missing	18		2.8		4	0.4
Self-reported Asthma	No	571		89.8		937	88.8
	Yes	63		9.9		107	10.1
	Missing	2		0.3		11	1.0
Self-reported shortness of	No	NA				939	89.0
breath	Yes	NA				103	9.8
	Missing					13	1.2
Asthma diagnosis	No	608		95.6		984	89.9
	Yes	19		3.0		71	6.7
	Missing	9		1.4		0	0.0
Asthma medication	No	586		92.1		973	92.2
	Yes	40		6.3		82	7.8
	Missing	10		1.6		0	0.0

NA = not available, i.e. questions not included in the questionnaire, Q_1_ = 1^st^ quartile, Q_3_ = 3^rd^ quartile.

1 Not available for pregnancies before 1982.

A Same sex female twin pairs born 1926–1966.

B Same sex female and male twin pairs born 1944–1971.

C Each twin was living together with a partner in a long-term relationship. The adolescent child was 11–22 years old and was living together with the parents. The cousins were the same sex and not more than 4 years apart in age.

There was some difference between the cohorts on distribution of the KSP variable somatic anxiety (higher ratings in study 1) but no difference for KSP psychic anxiety, whereas BAI was only measured in study 2. The two cohorts were also similar regarding distribution of the outcomes maternal and self-reported asthma, however asthma diagnosis in PAR was more common in study 2 (6.7%) than study 1 (3.0%), and similarly asthma medication in PDR was slightly more common in study 2 (7.8%) than study 1 (6.3%).

Table 2 displays the crude percentages of asthma outcomes by level of maternal anxiety. For levels of KSP somatic anxiety, there was no difference in the prevalence of asthma reported by the mother (low anxiety level 9.6%; moderate 8.4% and high 8.8% of asthma) or asthma reported by the child, with similar findings for KSP psychic anxiety. When maternal anxiety was assessed with BAI however, asthma reported by mother (low 6.5%; moderate 7.5% and high 12.3%) and child as well as breathlessness reported by child increased with increasing levels of anxiety. All scales (KSP somatic anxiety, KSP psychic anxiety and BAI) had increasing prevalence of breathlessness reported by the child when mothers reported increasing levels of anxiety. For the register-based outcomes there was a decreasing prevalence of asthma medication as mothers reported less KSP somatic and psychic anxiety.

**Table 2:** Percentage of reported asthma and breathlessness, register based asthma diagnosis and medication by level of reported maternal anxiety in a cross-sectional twin study.

Asthma indicator	Maternal anxiety scale	Level of anxiety		
		Lower	Moderate	Higher
		% with outcome		
**Outcomes reported in questionnaire:**				
Asthma reported by mother	Somatic anxiety	9.6	8.4	8.8
	Psychic anxiety	9.2	8.5	9.0
	BAI^1^	6.5	7.5	12.3
Asthma reported by child	Somatic anxiety	10.8	10.0	9.7
	Psychic anxiety	10.9	9.3	10.1
	BAI	9.7	9.4	11.5
Breathlesness reported by child^2^	Somatic anxiety	7.1	10.3	12.9
	Psychic anxiety	7.4	10.4	12.5
	BAI	7.8	9.0	12.9
**Outcomes from registers:**				
Asthma diagnosis	Somatic anxiety	4.9	5.5	5.5
	Psychic anxiety	6.2	5.0	4.7
	BAI	4.7	6.9	8.0
Asthma medication, twice^3^	Somatic anxiety	8.6	7.6	5.1
	Psychic anxiety	9.0	6.5	5.8
	BAI	7.8	6.4	8.6

1 Maternal anxiety assessed with BAI was only available for study 2.

2 Breathlessness reported by child was only available for study 2.

3 At least two purchases of asthma medication, except oral beta-2-agonists, on different days July 2005– December 2009.

Table 3 provides the corresponding crude and adjusted odds ratios with confidence intervals. KSP somatic or psychic anxiety was not significantly associated with maternal report of asthma in the child. However, there were significant associations between BAI and maternal report of asthma in the child (OR = 2.02; 95% CI 1.15–3.55). None of the maternal anxiety scales were significantly associated with asthma reported by child, but the highest levels of KSP somatic (OR = 1.74; 95% CI 1.04–2.91 and psychic (OR = 1.74; 95% CI 1.05–2.86) anxiety were significantly associated with breathlessness reported by the child. In line with child report of asthma, maternal anxiety was not associated with increased risk for the register-based outcomes of asthma diagnosis or asthma medication. In fact, there was an inverse association between KSP somatic anxiety and asthma medication (OR = 0.56; 95% CI 0.34–0.92). Further adjustment for the child’s own anxiety, as a potential mediator, resulted in very minor changes in the ORs. There was no statistically significant interaction between maternal anxiety and age of offspring. Smoking during pregnancy was available for a subset of the study population. Adjusting the analyses for this variable had a negligible effect on the estimated OR (results not shown).

**Table 3:** Crude and adjusted analyses of association between level of maternal anxiety and offspring asthma as reported in questionnaires and recorded in registers.

Asthma indicator	Maternal anxiety scale	Level	OR (95% CI)			
			Crude		Adjusted^4^	
**Outcomes reported in questionnaire:**						
Asthma reported	Somatic anxiety	Lower	1		1	
by mother	n = 1713/n = 1620^5^	Medium	0.86	(0.57; 1.29)	0.82	(0.54; 1.25)
		Higher	0.90	(0.58; 1.40)	0.81	(0.52; 1.27)
	Psychic anxiety	Lower	1		1	
	n = 1712/n = 1619^5^	Medium	0.93	(0.61; 1.40)	0.90	(0.59; 1.38)
		Higher	0.96	(0.63; 1.47)	0.94	(0.61; 1.46)
	BAI^1^	Lower	1		1	
	n = 1084/n = 1022^5^	Medium	1.15	(0.64; 2.06)	1.12	(0.61; 2.05)
		Higher	1.99	(1.16; 3.34)	2.02	(1.15; 3.55)
Asthma reported	Somatic anxiety	Lower	1		1	
by child	n = 1718/n = 1625^5^	Medium	0.92	(0.63; 1.35)	0.85	(0.57; 1.27)
		Higher	0.89	(0.59; 1.34)	0.81	(0.53; 1.22)
	Psychic anxiety	Lower	1		1	
	n = 1720/n = 1626^5^	Medium	0.84	(0.56; 1.24)	0.82	(0.54; 1.23)
		Higher	0.90	(0.61; 1.34)	0.87	(0.58; 1.30)
	BAI	Lower	1		1	
	n = 1076/n = 1016^5^	Medium	0.96	(0.58; 1.59)	0.89	(0.53; 1.52)
		Higher	1.21	(0.74; 1.97)	1.19	(0.72; 1.96)
Breathlesness	Somatic anxiety	Lower	1		1	
reported by child^2^	n = 1073/n = 1013^5^	Medium	1.48	(0.89; 2.46)	1.56	(0.93; 2.63)
		Higher	1.88	(1.14; 3.11)	1.74	(1.04; 2.91)
	Psychic anxiety	Lower	1		1	
	n = 1073/n = 1013^5^	Medium	1.45	(0.87; 2.41)	1.47	(0.86; 2.51)
		Higher	1.77	(1.08; 2.91)	1.74	(1.05; 2.86)
	BAI	Lower	1		1	
	n = 1075/n = 1014^5^	Medium	1.13	(0.67; 1.89)	1.10	(0.64; 1.86)
		Higher	1.66	(1.01; 2.74)	1.54	(0.91; 2.58)
**Outcomes from registers:**						
Asthma diagnosis	Somatic anxiety	Lower	1		1	
	n = 1670/n = 1628^5^	Medium	1.14	(0.67; 1.94)	1.08	(0.61; 1.89)
		Higher	1.13	(0.65; 1.97)	1.00	(0.57; 1.75)
	Psychic anxiety	Lower	1		1	
	n = 1672/n = 1629^5^	Medium	0.81	(0.49; 1.33)	0.84	(0.50; 1.42)
		Higher	0.74	(0.43; 1.25)	0.67	(0.39; 1.16)
	BAI	Lower	1		1	
	n = 1050/n = 1025^5^	Medium	1.48	(0.77; 2.83)	1.25	(0.64; 2.46)
		Higher	1.76	(0.91; 3.37)	1.66	(0.85; 3.22)
Asthma	Somatic anxiety	Lower	1		1	
medication^3^	n = 1669/n = 1627^5^	Medium	0.87	(0.56; 1.33)	0.84	(0.54; 1.32)
		Higher	0.58	(0.35; 0.95)	0.56	(0.34; 0.92)
	Psychic anxiety	Lower	1		1	
	n = 1671/n = 1628^5^	Medium	0.72	(0.46; 1.12)	0.70	(0.44; 1.10)
		Higher	0.63	(0.39; 1.01)	0.63	(0.38; 1.03)
	BAI	Lower	1		1	
	n = 1050/n = 1025^5^	Medium	0.80	(0.46; 1.40)	0.78	(0.43; 1.42)
		Higher	1.10	(0.63; 1.94)	1.18	(0.64; 2.13)

1 Maternal anxiety assessed with BAI was only available for study 2.

2 Breathlessness reported by child was only available for study 2.

3 At least two purchases of asthma medication, except oral beta-2-agonists, on different days July 2005– December 2009.

4 Adjusted for mother’s education, maternal smoking at time of the study, maternal asthma, sex and age of the child, preterm birth, birth weight and caesarean section.

5 Sample sizes for crude/adjusted analyses.

Table 4 summarises the children-of-twins analysis of the association between exposure from mother’s twin sister and outcome in the child in families where both twin mothers and their children responded (233 MZ pairs and 259 DZ pairs). For the analyses rendering statistically significant ORs between maternal anxiety and asthma outcome in Table 3, associations between anxiety of the mother’s twin sister and asthma outcome were of similar magnitude.

**Table 4:** Children of MZ +DZ twin mothers: Analyses of association between mother’s level of anxiety as well as her twin sister’s levels and asthma in the child as reported in questionnaires and recorded in registers.

Asthma indicator	Anxiety scale	Level	OR (95% CI) for exposure from				
			Mother		Mother’s twin sister		
**Outcomes reported in questionnaire:**							
Asthma reported	BAI^3,4^	Lower	1		1		
by mother	n = 954	Medium	1.64	(0.48; 5.61)	0.95	(0.28; 3.22)	
		Higher	4.96	(1.55; 15.80)	3.23	(1.11; 9.43)	
		Cont.	1.06	(1.01–1.12)	1.03	(0.98–1.08)	
Breathlessness	Somatic anxiety^4^	Lower	1		1		
reported by child^1^	n = 944	Medium	2.52	(1.01; 6.28)	1.64	(0.69; 3.90)	
		Higher	2.93	(1.09; 7.88)	2.85	(1.14; 7.11)	
		Cont.	1.06	(1.00–1.12)	1.08	(1.01–1.13)	
	Psychic anxiety^4^	Lower	1		1		
	n = 944	Medium	3.47	(1.22; 9.88)	1.11	(0.39; 3.13)	
		Higher	3.39	(1.24; 9.24)	1.40	(0.60; 3.24)	
		Cont.	1.06	(1.01–1.11)	1.05	(1.00–1.12)	
	BAI^3,4^	Lower	1		1		
	n = 945	Medium	1.91	(0.72; 5.06)	2.06	(0.75; 5.66)	
		Higher	2.99	(1.15; 7.77)	3.87	(1.39; 10.80)	
		Cont.	1.03	(0.99–1.07)	1.06	(1.02–1.11)	
**Outcomes from registers:**							
Asthma	Somatic anxiety	Lower	1		1		
Medication^2^	n = 1248	Medium	1.46	(0.79; 2.68)	1.10	(0.58; 2.06)	
		Higher	0.62	(0.31; 1.28)	0.93	(0.48; 1.80)	
		Cont.	0.96	(0.90–1.01)	1.01	(0.96–1.06)	

1 Breathlessness reported by child was only available for study 2.

2 At least two purchases of asthma medication, except oral beta-2-agonists, on different days July 2005– December 2009.

3 Maternal anxiety assessed with BAI was only available for study 2.

4 Spearman’s correlation coefficient between twin sisters (continuous scale) is 0.30 for BAI; 0.33 for Somatic anxiety; 0.34 for Psychic anxiety.

All analyses adjusted for mother’s education, maternal smoking at time of the study, maternal asthma, sex and age of the child, preterm birth, birth weight and caesarean section.

## Discussion

In line with previous studies, we found some evidence for an association between reported maternal anxiety and childhood asthma and significant associations between maternal anxiety and breathlessness reported by the child. This indicates that the associations are not only due to rater bias. However we found no positive association between maternal anxiety and register-based measures of asthma diagnosis or medication. The findings in the children-of-twins design indicate that previous studies on maternal anxiety and maternal reported childhood asthma [2], [7], [13], [14] may have been caused by familial confounding, i.e. genetic and environmental factors shared by the twins.

The novelty of this study is the children-of-twin analyses, which makes it possible to investigate if the association between maternal anxiety and adolescent outcome is related to familial (shared genetic and environmental) factors. For the analyses rendering statistically significant ORs between maternal anxiety and offspring asthma outcome, most were comparable to those found between aunts’ anxiety level and their niece’ or nephews’ asthma outcomes. Thus our results, based on the largest twin study on maternal anxiety and outcomes in adolescence suggest that the associations are due to familial factors rather than a specific effect of maternal anxiety. A likely candidate for this familial confounding would be heritable personality traits associated with anxiety, even though we could not exclude shared environmental confounding due to lack of power. It is however unlikely that shared environmental factors are involved since most behavioural genetic studies that have examined anxiousness have found little evidence of shared environmental contributions to these personality characteristics [26]. As a consequence, it is likely that associations between asthma and anxiety are explained by genetic factors. Future studies with larger samples of MZ twins and their children should be able to clarify the distinctions between influences of genetics versus environmental factors common to the adult twin mothers.

Maternal anxiety measured by self-report [7] or clinical diagnosis [12] and its association with offspring asthma has been the subject of several studies, mainly with focus on the effect during the early months in life [12], [13], [27], [28] whereas studies on adolescents and young adults has had focus on the relationship between anxiety and asthma in the individual [11]. Genes associated with asthma seem to be susceptible to different types of environmental influence for their onset [29]. A recent study also identified a locus specific to childhood asthma [30]. Little is known about how environmental stress influences older children. Our findings provide one step in remedying this shortcoming by adding substantially to existing knowledge. An alternative finding related to breathlessness reported by the adolescent child rather than objective asthma, however, introduces the possibility that children with subclinical asthma present symptoms when exposed to stress, or that the breathlessness is related to symptoms of anxiety such as shortness of breath, panic attack or heavy breathing.

The strength of this study is the large sample size of mothers with adolescent children, a high response rate and well validated scales from questionnaires linked to the Swedish Medical Birth Registry, Prescribed Drug Register and National Patient Register, along with good control of measures and confounders. Sweden offers free inpatient and outpatient medical care to all residents. The National Patient Register covers inpatient and outpatient visits, but not visits to general practitioners and therefore we included asthma medication use from the Prescribed Drug Register as an additional asthma ascertainment strategy in the present analyses. By using these asthma ascertainment criteria, we believe that we were able to identify the vast majority of asthma cases that had come to the notice of the health care system. An additional strength is the extended children-of-twin design which contributes to the genetic and environmental aspects of associations.

Although this study has been thoroughly designed to answer the question of maternal anxiety and the influence on adolescent asthma, a few limitations should be observed. The asthma questions related to previous six months, so there is a risk that not all children with asthma have been identified in the two cohorts. Also, the TOSS study had a cross-sectional design, which makes it more difficult to analyse causal direction of the associations. Linkage to the national health registers allows questionnaire report of the exposures to be followed by objective outcomes of asthma medication and hospital visits.

In TOSS, single parent women were excluded, which results in a slightly higher socioeconomic status in the TOSS sample than in Sweden overall [31]. We investigated the relationship between maternal anxiety and report of asthma in the adolescent child. Hence, generalising to a female population does seem reliable. The twin study approach has an established generalizability [32] and the use of spouses in this study bridges the risk of limitations in a twin study.

This study illustrates the need of further studies to clarify whether it is genetic or familial environmental confounding that explains the correlation between maternal anxiety and respiratory outcomes. Such a study should preferably have a longitudinal perspective and take all known aspects of asthma into consideration. Combined with aspects of changes in gene expression, and a clearer understanding of the genetics of asthma, this can facilitate attempts to suggest future public health interventions.

In conclusion, the maternal report of recent anxiety is significantly associated with some aspects of maternal report of asthma and the child’s report of breathlessness but not asthma medication in her child. The increased risk of asthma in relation to anxiety seems to be related to familial (genetic or maternal familial environment) confounding rather than an effect of the individual environment.
